# The neuromodulatory fragility hypothesis of Alzheimer's disease pathogenesis

**DOI:** 10.1002/alz.71249

**Published:** 2026-03-09

**Authors:** Alfie Wearn, Kate M. Onuska, Taylor W. Schmitz, Gary R. Turner, R. Nathan Spreng

**Affiliations:** ^1^ Department of Neurology and Neurosurgery Montreal Neurological Institute, McGill University Montreal Quebec Canada; ^2^ Department of Physiology & Pharmacology Western Institute for Neuroscience, Western University London Ontario Canada; ^3^ Department of Psychology York University Toronto Ontario Canada; ^4^ McConnell Brain Imaging Centre McGill University Montreal Quebec Canada; ^5^ Douglas Mental Health University Institute—Research Center Verdun Quebec Canada; ^6^ Department of Psychiatry McGill University Montreal Quebec Canada

**Keywords:** acetylcholine, Alzheimer's disease, amyloid beta, dopamine, neuroinflammation, neuromodulatory subcortical systems, neuronal resilience, neuronal vulnerability, noradrenaline, orexin, pathogenesis, risk factors, serotonin, tau

## Abstract

Sporadic Alzheimer's disease (AD) is associated with numerous risk factors, yet its precise cause remains unclear. Here, we describe a novel framework for AD pathogenesis, whereby diverse risk factors converge on neuromodulatory subcortical systems to confer AD risk or resilience. Neuromodulatory projection neurons are uniquely fragile due to their large size, sparse myelination, and high basal metabolic demands. We propose that the increased prevalence of AD in older adult populations likely reflects a universal weakness within these projection systems, which is increasingly exposed as cellular transport and maintenance mechanisms deteriorate with age. The key insight of this “neuromodulatory fragility hypothesis” is that neuromodulatory system dysfunction is sufficient to explain both tau hyperphosphorylation and amyloid beta plaque formation, the two pathological hallmarks of AD. We therefore predict that strengthening or preserving the endogenous functions of these systems in midlife represents the most effective strategy for preventing AD.

## Highlights

- We describe a neuromodulatory fragility hypothesis for Alzheimer's disease (AD) pathogenesis.
- Neuromodulatory neurons are extremely fragile due to high metabolic demands.
- Their early dysfunction leads to downstream tau misfolding and amyloid beta (Aβ) pathology.
- Eventual Aβ–tau interactions cause rapid spreading of pathology and dementia onset.
- Improving neuromodulatory resilience presents the best dementia‐prevention strategy.

## 1 INTRODUCTION: WHAT CAUSES ALZHEIMER'S DISEASE?

Identifying the cause of Alzheimer's disease (AD) remains one of the most urgent challenges in modern medicine, yet its complexity continues to preclude a single parsimonious explanation of pathogenesis.^1^ Many risk factors have been identified, which combine and interact to confer relative risk or resilience.^2^ These include age, genetics, traumatic brain injury, comorbidities, physical inactivity, sleep disruption, and viral or bacterial infection, among others.^1^, ^2^ The diversity of these “causes” contrasts sharply with the relatively uniform sequence of pathological events that define AD. While individual progression patterns and clinical presentations can vary^3^, ^4^ (discussed in section 8), AD typically unfolds in the brain as follows: tau neurofibrillary tangles and pre‐tangles spread according to Braak stages,^5^ amyloid beta (Aβ) plaques emerge according to Thal stages,^6^ and subsequent tau‐associated neurodegeneration drives canonical patterns of cognitive decline, ultimately leading to dementia.^7^ A system whereby varied causes lead to relatively uniform outcomes implies the existence of a single upstream vulnerability: a common breakpoint upon which diverse insults converge to initiate a pathological cascade (Postulate 1, Box 1). Furthermore, by Occam's razor, a single breakpoint is much more likely an explanation of pathogenesis than multiple simultaneously dysfunctioning systems (Postulate 2, Box 1). This is especially true in a disorder as common as AD, which affects one third of people over the age of 85.^8^ There is, therefore, a need for explanations of AD pathogenesis that unify the emergence of both Aβ and tau pathology under a single cause. Finally, given the high prevalence of AD, the single breakpoint is likely a feature of normal brain architecture rather than an obscure abnormality (Postulate 3, Box 1). Identification of this breakpoint is critical, as its protection would provide the most effective option for disease prevention.^9^

We argue that neuromodulatory subcortical projection neurons represent a prime candidate for this vulnerable breakpoint. These neurons are uniquely fragile: their large, sparsely myelinated, tau‐rich axons have extraordinary energy demands, making them highly dependent on a steady supply of resources and fully functional repair mechanisms.^10^, ^11^ We propose that the heightened prevalence of AD among older adults therefore reflects a universal weakness within these projection systems, which is increasingly exposed in older age when cellular transport and maintenance mechanisms falter.^12^, ^13^ In a sentence: we believe that it is no coincidence that the hardest‐to‐maintain parts of the most fragile neurons are enriched for a pre‐pathogenic component (tau) of the most common cause of dementia.

**FIGURE 1:**
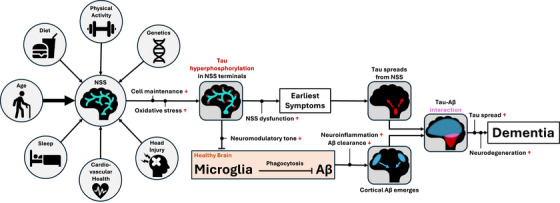
Flowchart of the proposed neuromodulatory fragility hypothesis of AD pathogenesis. Risk factors confer vulnerability or resilience to NSSs either directly or indirectly through disproportionate consequences in whole brain health. Due to disruptions in cell maintenance in distal axons, tau becomes hyperphosphorylated and misfolded, causing axonal instability and reduced cortical neuromodulatory tone. This causes nebulous early symptoms (sleep disruption, mood instability, attention deficits) and disrupts microglial homeostasis, impairing amyloid clearance. Misfolded tau spreads transneuronally from NSS cells, while Aβ appears in increasingly widespread cognitive regions. When Aβ and tau interact (typically in inferior temporal cortex), tau spread, and associated neurodegeneration are exacerbated, and cognition declines rapidly, eventually leading to dementia. Aβ, amyloid beta; AD, Alzheimer's disease; NSS, neuromodulatory subcortical system.

We propose the following mechanistic framework whereby AD, from its initial pathogenesis to clinical dementia, can be understood as a downstream consequence of neuromodulatory fragility (Figure 1). Declining metabolic efficiency and impaired cellular transport and clearance mechanisms in advancing age disproportionately affect neuromodulatory neurons, particularly in tau‐rich axons extremely distal from the cell nucleus. Accumulated oxidative stress drives tau hyperphosphorylation and misfolding, leading to axonal instability and reduced neuromodulatory tone at cortical endpoints. This loss of neuromodulatory input disrupts microglial homeostasis and impairs the clearance of early Aβ species, eventually leading to plaque formation. Misfolded, hyperphosphorylated tau spreads slowly and transneuronally through highly active networks, while Aβ plaques continue to form across the cortex. Eventual local interactions of Aβ and tau pathology accelerate the activity‐dependent transneuronal spread of tau due to Aβ‐induced hyperexcitability, culminating in widespread neurodegeneration and dementia onset.

In this perspective paper we describe this “neuromodulatory fragility hypothesis” of AD pathogenesis in more detail, describing a broad view of the entire AD process from pathogenesis to dementia onset. The framework this hypothesis provides shifts the focus from late‐stage cortical hallmarks to vulnerable subcortical origins which become dysfunctional long before the clinical manifestation of the disease. The framework situates sporadic AD as a disorder of statistical vulnerability rather than a singular initiating insult per se. In lieu of refuting the amyloid hypothesis, which has dominated thinking and research on AD for > 30 years,^14^ we suggest a reframing, whereby Aβ accumulation may be a downstream consequence of a fundamental dysfunction of neuromodulatory subcortical projection neurons, rather than the initiating trigger of sporadic AD pathogenesis. By viewing AD through the lens of neuromodulatory fragility, we can integrate disparate findings on tau seeding, the neuroinflammatory response, and nebulous prodromal behavioral changes into a unified theory of AD pathogenesis.

**BOX 1: Postulates of the neuromodulatory fragility hypothesis of Alzheimer's disease pathogenesis**

Postulate 1: Diverse causes leading to (relatively) uniform outcomes suggests a single upstream vulnerability.

Postulate 2: A single breakpoint is a simpler explanation of pathogenesis than multiple breakpoints, especially in an extremely common disorder (Occam's razor).

Postulate 3: In an extremely prevalent disorder (like Alzheimer's disease [AD]), the breakpoint is more likely a feature of normal brain architecture than an obscure abnormality.

Neuromodulatory subcortical projection neurons, specifically their tau‐rich axonal terminals, are a prime candidate for this single vulnerable breakpoint. Multiple risk factors can converge on this system to confer risk of AD. Variation across individuals in neuromodulatory resilience is a likely predictor of relative risk for AD.

## 2 NEUROMODULATORY PROJECTION NEURONS ARE A SYSTEM‐WIDE WEAK POINT

Neuromodulatory subcortical systems can be analogized as the conductors to the orchestra of the brain. Where glutamatergic and GABA‐ergic neurons facilitate the bulk of the brain's activity (the orchestra), dramatically smaller populations of neuromodulatory neurons (the conductors) shape the dynamics and characteristics of their combined activity.^15^, ^16^ These systems release neurotransmitters such as noradrenaline, acetylcholine, dopamine, serotonin, and orexin. Given their pivotal role in modulating brain activity, these neuromodulators are implicated in many fundamental cognitive and behavioral processes including regulating sleep–wake cycles, mood stability, attention, appetite control, even interacting with the brain's immune system.^17^, ^18^, ^19^, ^20^, ^21^, ^22^

A key distinction between neuromodulatory neurons and other projection neurons is their use of “volume transmission”: they can release neurotransmitters from varicosities along the axon into the extracellular space without a specific postsynaptic target, in addition to classical synaptic release.^23^ Several anatomical and physiological features simultaneously enable this mode of signaling while simultaneously heightening their fragility:

1. **Extreme axonal length and arborization**: The projection neurons of neuromodulatory systems originate from small clusters of cell bodies (e.g., locus coeruleus, nucleus basalis of Meynert, dorsal raphe, ventral tegmental area, lateral hypothalamus), yet send vast, highly arborized projections across the entire neocortex. The extreme length and breadth of these combined arborizations allows each neuron to modulate large areas.^24^, ^25^, ^26^ However, maintaining such long, branched projections is metabolically expensive. These systems require continuous transport of proteins, mitochondria, and other cellular components, rendering them chronically dependent on reliable access to nutrients, growth factors, and oxygen, as well as effective waste clearance and repair mechanisms.^27^, ^28^
2. **High tonic firing rates**: To sustain basal neuromodulatory tone over large target areas, neuromodulatory neurons exhibit high tonic firing rates, with regular transient bursts during behaviorally relevant events.^29^, ^30^, ^31^, ^32^ This firing pattern is well suited to facilitate slow, diffuse modulation via volume transmission, but further amplifies metabolic demand, increasing susceptibility to any disruption in oxidative balance, calcium homeostasis, or energy production.
3. **Poor myelination**. Neuromodulatory axons are typically thin and only sparsely myelinated. This facilitates “leakiness” of neurotransmitter along the axon's length—the definitive feature of “volume transmission.” However, the lack of myelination and thin caliber of these neurons offers little protection against oxidative or mechanical injury.^33^, ^34^ Cortical regions with higher myelination are relatively resistant to pathology and neurodegeneration, suggesting that myelin may represent a substrate of “brain reserve” that these projection systems largely lack.^34^, ^35^ The constant axonal remodeling that may be required to maintain these delicate projections could further exacerbate the metabolic demands of these systems.

Taken together, the features that allow neuromodulatory systems to modulate large‐scale brain states (extreme arborization, high tonic activity, poor myelination) also impose high energetic demands, low tolerance for metabolic and oxidative stress, and a strong propensity to accumulate damage over the lifespan. The fact that these same features are essential for their function helps explain the apparent evolutionary maladaptiveness of these features: typical evolutionary routes to increase resilience, such as increasing myelination or pruning excess branches, would directly impair their modulatory capacity and are therefore unlikely to be favored by natural selection.

Aging, the primary risk factor for sporadic AD, is poised to disproportionately affect these low‐tolerance systems through changes such as mitochondrial dysfunction, oxidative stress, and disrupted calcium homeostasis.^11^, ^12^, ^36^ Monoaminergic neurons are particularly at risk for oxidative stress. By‐products of noradrenaline and dopamine synthesis generate reactive oxygen species throughout life, particularly in axons and terminals where these neurotransmitters are produced.^37^ Neuromelanin, a compound found in monoaminergic neurons, normally sequesters these toxic metabolites, but when its capacity is reached in older age, intracellular toxicity rises sharply, possibly marking a transition from resilience to degeneration.^38^ In cholinergic neurons, age‐related reductions in calcium‐binding proteins increase their susceptibility to calcium‐induced excitotoxicity and pathological processes.^39^ Aging therefore imposes a critical bottleneck on these inherently fragile neuronal systems.

Other risk factors for AD likely modulate the vulnerability of neuromodulatory projection neurons, either through specific direct mechanisms or through systemic changes to brain health. Take as an example the apolipoprotein E (APOE) ε4 gene, the single largest genetic risk factor for sporadic AD. Carriers of one or two *APOE* ε4 alleles face up to 3‐ or 15‐fold greater risk, respectively, than *APOE* ε3 homozygotes.^40^ *APOE* ε4 is thought to impair myelin regulation.^41^ It is feasible that myelin dysregulation would disproportionately increase the vulnerability of neuromodulatory projection neurons which already lack robust myelination and therefore have little to no redundancy on which to fall back. In effect, we suggest that what little protection these neurons have under an *APOE* ε3 genotype is weakened or lost with an *APOE* ε4 genotype, further destabilizing an already fragile system. The inverse may well be true of the neuroprotective *APOE* ε2 allele. This framework aligns with evidence of selective neuromodulatory system damage in demyelinating conditions such as multiple sclerosis and encephalomyelitis.^42^

We have so far explained how upstream risk factors converge upon neuromodulatory systems to cause disproportionate dysfunction. The next sections will explain how this dysfunction is sufficient to explain the downstream emergence of the Aβ and tau pathology which define AD.

## 3 TAU HYPERPHOSPHORYLATION IS A DIRECT OUTCOME OF NEUROMODULATORY SYSTEM DISRUPTION

**FIGURE 2:**
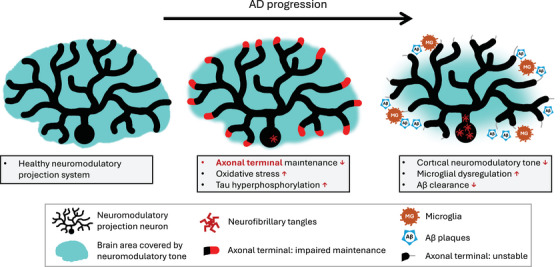
Schematic depiction of the emergence of Aβ plaques and tau tangles due to neuromodulatory dysfunction. The left image depicts a healthy neuromodulatory projection system with good neuromodulatory tone across the entire neocortex. The middle panel depicts the emergence of tau dysfunction due to the convergence of lifetime‐accumulated risk factors. This dysfunction (shown in red) occurs first in the most distal, and therefore most vulnerable, branches of the axonal tree. The image also depicts the emergence of tau tangles in the cell bodies. The right panel shows how regions where tau was disrupted become destabilized and dysfunctional (depicted by thin gray lines in the place of red areas in the middle panel). This instability gives rise to reduced neuromodulatory tone, microglial dysregulation, and impaired clearance of Aβ oligomers, resulting in the emergence of Aβ plaques. At this stage, tau tangles are abundant in the neuromodulatory neuron cell bodies. Aβ, amyloid beta; AD, Alzheimer's disease.

Tau is a protein that binds and stabilizes microtubules, supporting axonal structure.^43^ In the highly arborized projection neurons of neuromodulatory systems, axons are extremely distal from the cell nucleus.^24^, ^25^, ^26^ This makes their axonal machinery, of which physiological tau is a key component, particularly susceptible to disruptions in transport, repair, and clearance processes.^44^, ^45^, ^46^ In other words, these axons, especially distal, tau‐rich regions, are the most vulnerable part of an already fragile system (Figure 2).

Reliable clearance of reactive oxygen species from these distal structures is particularly critical. When reactive oxygen species accumulate (e.g., as by‐products of the monoaminergic synthesis pathway^38^, ^47^), oxidative stress triggers tau hyperphosphorylation, producing misfolded forms of tau that can no longer bind and stabilise microtubules.^48^ The result is axonal instability, disrupted intracellular transport, and impaired neurotransmission.^44^, ^45^

Misfolded oligomers of hyperphosphorylated tau cluster together to form neurofibrillary tangles (and pre‐tangles), which are first detectable in neuromodulatory nuclei in presymptomatic AD.^10^, ^49^ Tau pre‐tangles are reported as early as the second decade of life in the locus coeruleus,^18^, ^50^ and the third decade of life in the nucleus basalis of Meynert.^51^ Tangles have also been found in orexinergic neurons of the lateral hypothalamus^52^ and serotonergic dorsal raphe neurons^53^, ^54^ in presymptomatic AD. In the locus coeruleus and basal forebrain, hyperphosphorylated tau species have been shown to lead to decreased fiber density and reduced neuromodulatory tone.^48^, ^55^

A growing body of evidence supports the idea that tau pathology spreads transneuronally in an activity‐dependent manner, progressing most rapidly along densely connected pathways.^56^, ^57^ Early propagation likely occurs between neuromodulatory nuclei themselves, given their extensive inter‐connectivity, which, incidentally, may complicate efforts to identify the precise initial locus of pathology in population‐based studies. Knowledge of strong connections between these nuclei and cortical sites of early tau pathology in AD, together with evidence that tau pathology and atrophy in neuromodulatory nuclei precede corresponding changes in medial temporal regions,^58^, ^59^, ^60^, ^61^, ^62^ have led to the proposal that tau advances from subcortical hubs to the medial temporal lobe and beyond, following canonical Braak staging.^5^ Under the neuromodulatory fragility hypothesis, neuromodulatory nuclei act as seed points, propagating tau pathology to densely connected efferents to initiate cortical pathology. While tau accumulation in these early stages causes local neurodegeneration, the extent of spread and, by extension, overt clinical symptoms, remain limited until the process is amplified by Aβ pathology (described in section 5).^63^

## 4 NEUROMODULATORY SYSTEM DEGRADATION DISRUPTS Aβ CLEARANCE THROUGH NEUROINFLAMMATORY PATHWAYS

Interactions between neuromodulatory systems and Aβ are less well characterized than those with tau. The link is perhaps less obvious as Aβ plaques rarely accumulate around neuromodulatory cell bodies until relatively late stages of AD, typically Thal stage 5.^6^ Nevertheless, several mechanisms suggest that neuromodulatory system dysfunction could underlie the emergence of Aβ pathology.

As tau hyperphosphorylation destabilizes the axons of neuromodulatory projections (as described in section 3), distal axons degenerate, and cortical neuromodulatory tone declines^64^ (Figure 2). Reduced neuromodulatory tone leads to the release of pro‐inflammatory cytokines and microglial dysregulation, which, in turn, impairs Aβ clearance.^65^, ^66^, ^67^, ^68^ Experimentally induced degeneration of cholinergic and noradrenergic subcortical systems in animal models leads to Aβ plaque accumulation.^65^, ^69^ Conversely, replacement or receptor‐agonist treatment can reverse these downstream effects, supporting a causal role for neuromodulatory dysfunction in promoting Aβ deposition.^70^, ^71^ While most direct evidence for the neuroinflammation‐mediated dysregulation of Aβ clearance concerns the noradrenergic and cholinergic systems, similar anti‐inflammatory and neuroprotective properties have been observed for orexin.^72^

Neuromodulatory neurons may also contribute directly to early Aβ seeding. Both noradrenergic and cholinergic subcortical neurons have been shown to accumulate intracellular Aβ oligomer species in neurites and axon terminals.^73^, ^74^ Furthermore, noradrenaline may directly facilitate amyloidogenic processing of the amyloid precursor protein.^75^, ^76^ When clearance mechanisms are performing optimally, early Aβ species have little chance to cluster into plaques. However, once microglial dysfunction arises, Aβ fails to be cleared, and may therefore act as an initial seed that facilitates Aβ plaque formation, as described above. Aβ species may also induce or exacerbate hyperexcitability of neuromodulatory systems,^77^ which is likely to cause further metabolic strain and oxidative stress, augmenting downstream pathological processes.

In contrast to the transneuronal spread of tau, Aβ plaque formation appears wherever distal neuromodulatory tone is depleted. As with leaves falling from an autumn tree, the exact branch to lose foliage first may differ between individuals of even similar trees, but loss is inevitable once environmental conditions shift, and the tree restricts nutrients to more proximal regions. This analogy aligns with the observed interindividual variability in Aβ plaque localization in early Thal stages,^6^ despite the high prevalence of Aβ among even cognitively unimpaired older adult populations.^78^

Neuroinflammation is a critical but poorly understood element of AD, often debated as either a cause, effect, or epiphenomenon of the disease process. The neuromodulatory fragility hypothesis highlights a specific role for neuroinflammation in triggering and amplifying spread of Aβ pathology. The positive feedback loops by which neuroinflammation shifts from neuroprotection to neurotoxicity amplify the difficulty of studying the exact causative role of neuroinflammation in AD. Our model clarifies the sequence of events, which will aid generation of specific testable hypotheses to better understand this complex relationship.

## 5 TAU MISFOLDING PRECEDES PLAQUE ACCUMULATION BUT IS SLOW IN THE ABSENCE OF Aβ

Neurodegeneration and symptom onset correlate more strongly with tau neurofibrillary tangles than with Aβ pathology.^79^ However, in the absence of Aβ, tau‐associated toxicity, behavioral impairments, and the spatial extent of tau are greatly attenuated in AD.^80^ This latter detail likely reflects the interaction of two key mechanisms driven by the AD hallmarks: Aβ causes neuronal hyperexcitability, and tau spreads transneuronally in an activity‐dependent manner.^63^, ^81^

In our neuromodulatory fragility hypothesis of AD pathogenesis, tau misfolding originates in neuromodulatory projection neurons and spreads slowly along dense, highly active pathways, most notably projecting from neuromodulatory nuclei to the medial temporal lobe. In the absence of Aβ, as in primary age‐related tauopathy, tau remains spatially restricted but still colocalizes with neurodegeneration.^82^ In AD, as both tau and Aβ pathologies spread, they eventually converge. At this convergence point, significant Aβ‐driven neuronal hyperexcitability amplifies transneuronal tau propagation, driving rapid global distribution of tau. Graph theory and network modeling studies support this model, identifying the inferior temporal lobe as the most common site of Aβ–tau colocalization.^83^, ^84^ This colocalization and subsequent increasing rate of tau propogation is likely the tipping point between presymptomatic and symptomatic disease.

Because tau spread and symptom severity depend on Aβ pathology, Aβ is sometimes regarded as an “upstream” disease feature relative to altered tau proteostasis.^80^ However, our model positions tau dysregulation as the earliest molecular consequence of neuromodulatory system failure, preceding Aβ pathology. These seemingly contradictory views are reconcilable when considered across scales. With a traditional systems‐level view of disease, focusing on clinical symptom onset, Aβ moderates the spread and severity of tau pathology, and hence is considered “upstream”.^80^, ^81^ However, as we have described in the previous sections, at the cellular level, resource disruption in fragile neuromodulatory systems provides the initiating conditions that trigger tau misprocessing, which is sufficient to explain Aβ accumulation through microglial dysregulation.^45^, ^48^, ^64^, ^65^ It is clear that these views are not in fact contradictory; rather, they prioritize different scales at which the term “upstream” is defined. The idea that tau misfolding precedes that of Aβ is not new, but the two processes are typically regarded as independent inciting events.^85^, ^86^ In contrast, our framework positions neuromodulatory fragility as a single upstream cause for both.

In humans, Aβ and tau interact continuously throughout AD. Pathogenic tau species can promote Aβ deposition (as discussed above), while Aβ may directly augment tau hyperphosphorylation for example through GSK‐3β signaling.^37^ Direct injection of Aβ can initiate tau pathology in rodent models expressing mutant human tau,^87^ but we argue that this injection “skips ahead” in the usual pathological progression, bypassing the neuromodulatory dysfunction‐associated altered tau proteostasis and triggering the positive feedback loop from an alternative entry point. In other words, this rodent model, while useful for studying downstream stages of the disease, does not recapitulate natural pathogenesis of sporadic AD.

This mechanism may, however, align with pathogenesis in autosomal dominant AD. Autosomal dominant AD is a rare genetic form of AD characterized by early symptom onset, and is caused by mutations in three genes which directly affect the Aβ pathway.^88^ While the clinical presentation and late‐stage pathology of sporadic AD versus autosomal dominant AD are very similar, it remains unclear to what extent their upstream mechanisms of pathogenesis differ. We speculate that the intrinsic fragility of neuromodulatory projection neurons is a shared feature of pathogenesis in both cases, with differences arising in the balance and timing of misfolded Aβ and tau species. Indeed, early vulnerability of noradrenergic,^89^, ^90^ cholinergic^91^ (but see Teipel et al.^92^), and serotonergic^93^ systems has been demonstrated in individuals carrying autosomal dominant AD mutations. We propose that whereas in sporadic late‐onset AD, toxic Aβ oligomers are efficiently cleared until older age (e.g., by proteases and microglia), autosomal dominant AD mutations create conditions of chronic Aβ overproduction or impaired clearance that overwhelm these mechanisms much earlier in life, effectively ensuring earlier onset of the same neuromodulatory fragility‐driven cascade.

In our neuromodulatory fragility hypothesis, we highlight how a single point of fragility can trigger a self‐perpetuating state of pathology. Closely interacting factors can rapidly obscure the direction of cause–effect relationships in experimental settings. Mechanistic explanations such as those presented here by the neuromodulatory fragility hypothesis are therefore crucial for forming effective research questions and hypotheses aimed at disentangling these relationships.

## 6 EARLY BEHAVIORAL SYMPTOMS ARE CONSISTENT WITH EARLY NEUROMODULATORY DYSFUNCTION

We acknowledge that compensatory mechanisms (potentially within these neuromodulatory systems) maintain near‐normal cognitive functioning for many years, despite concurrent pathophysiological processes.^94^ Thus, clinically detectable symptoms most likely emerge as these mechanisms reach their limits and begin to fail. In AD, an episodic memory deficit is the most definitive and clinically recognized early symptom; however, patients often report symptoms months or years before measurable memory impairment.^95^ These subtle perturbations in cognition and behavior align closely with a model of early neuromodulatory system dysfunction, including: dysregulation of sleep–wake cycles (orexin, noradrenaline), attention deficits (acetylcholine, noradrenaline), and mood instability (serotonin).^10^, ^95^ The emergence of neuropsychiatric symptoms in later disease stages likely reflects a more substantial breakdown of global neuromodulatory control.^96^

## 7 PREDICTIONS OF THE NEUROMODULATORY FRAGILITY HYPOTHESIS

The proposed framework makes several testable predictions about AD that could guide future research. A key prediction of the hypothesis is that enhancing the resilience of neuromodulatory systems should significantly delay or prevent the onset of AD. Strengthening these systems, through interventions that reduce oxidative stress or support intracellular transport and repair, could slow or halt early pathogenesis if applied early enough. Rather than proposing a single “cure‐all” mechanism, this framework aligns with existing evidence on the importance of modifiable risk factors such as cardiovascular health, sleep regulation, and protection from head injury as effective preventive strategies in midlife.^2^ That said, a theoretical pharmacological augmentation of neuromodulatory resilience would provide effective targeted AD protection.^9^

The neuromodulatory fragility hypothesis also predicts that accurate tracking of neuromodulatory system health could improve early detection and monitoring of disease progression. Recent advances in neuroimaging, including quantitative and neuromelanin‐sensitive magnetic resonance imaging, ultra‐high‐field diffusion imaging, and positron emission tomography, have improved in vivo assessment of these systems.^97^, ^98^, ^99^ As these tools mature and become more widely available, clinical monitoring of neuromodulatory integrity may become feasible for early diagnosis and therapeutic evaluation.

An additional implication of the model concerns the limited efficacy of anti‐Aβ treatments, especially if applied late in the disease process.^85^ Once cortical Aβ burden is high, positive feedback loops characteristic of AD are already established, and the spreading of tau pathology is already well underway. Removing Aβ at this stage may modestly slow disease progression but is unlikely to halt it. While even incremental slowing of disease remains valuable (if side effects can be minimized) we argue that therapies targeting patients who are already Aβ positive may miss the critical early window of intervention. According to the neuromodulatory fragility hypothesis, Aβ is positioned as an important but downstream element of AD pathology, while preventing or mitigating neuromodulatory dysfunction offers a more promising means of arresting AD progression.

## 8 UNANSWERED QUESTIONS

While the neuromodulatory fragility hypothesis ties together many known features of the AD trajectory, several questions remain open and warrant further investigation.

One such question concerns atypical subtypes of AD. It is becoming increasingly apparent that although progression patterns of tau are often categorized into canonical Braak stages, there is evidence for multiple subtypes of tau progression pattern, each with subtly different characteristics in terms of severity, topography, speed of progression, and behavioral presentation.^3^, ^4^ It remains unclear whether all AD subtypes share the same underlying etiology. Tau transmission along neuromodulatory projection pathways may follow probabilistic routes that vary across individuals leading to different clinical and anatomical presentations. Alternatively, different subtypes might arise from dysfunction in distinct neuromodulatory systems or through entirely separate mechanisms.

A related question is whether all neuromodulatory systems are equally vulnerable or whether specific nuclei are consistently affected first. If the latter is true, targeted strategies to augment resilience of a given, particularly vulnerable, neuromodulatory system could be highly effective. Alternatively, neuromodulatory systems may be strongly interdependent, and dysfunction in any one of a few candidate systems may be swiftly transmitted to others, necessitating a broader therapeutic strategy.

Additionally, while we have proposed that environmental and demographic factors confer risk of AD through their impact on neuromodulatory systems, the specific underlying mechanisms remain poorly understood. For example, AD is more prevalent in women, possibly reflecting menopause‐related neuroendocrine changes that increase neuromodulatory fragility.^100^ Likewise, differences in disease incidence and clinical presentation across ethnic groups highlight the need to clarify how genetic and sociocultural factors interact with brain resilience and neuromodulatory system health.^101^

Finally, the proposed neuromodulatory fragility hypothesis must address the question of why AD appears so human specific, despite neuromodulatory systems being relatively well conserved across species. Non‐human primates do develop Aβ pathology, but tau burden diminishes rapidly with increasing phylogenetic distance from humans.^102^ Viewed through the lens of the neuromodulatory fragility hypothesis, this pattern suggests we should look closely at species differences in neuromodulatory systems. Many mammals express neuromelanin in monoaminergic nuclei (e.g., locus coeruleus, substantia nigra) but typically at much lower levels than humans.^103^ Even across primates, neuromelanin content is greater in species more closely related to us.^103^ A striking exception, in terms of both the expression of AD‐like pathology and neuromelanin, can be seen in certain toothed whales (odontocetes).^104^, ^105^ In contrast to even our closest evolutionary cousins, odontocetes have been observed exhibiting human‐like co‐occurrence of Aβ plaques and tau pathology^104^ as well as neuromelanin in the locus coeruleus with ultrastructural features remarkably similar to that of humans.^105^ Although direct quantitative comparisons across primates and odontocetes are still lacking, this apparent qualitative correlation between neuromelanin and AD‐like pathology raises the hypothesis that neuromelanin burden may serve as a cross‐species marker of cumulative neuromodulatory strain, which facilitates the emergence of AD.

The origin of this putative species‐specific neuromodulatory strain remains unclear. One possibility is that a dramatically expanded neocortex provides unusually large target fields that must be modulated by relatively small populations of neuromodulatory cells. Indeed, human locus coeruleus and nucleus basalis of Meynert contain far fewer neurons relative to neocortical volume compared to non‐human primates,^106^, ^107^ a pattern that may hypothetically extend to odontocetes which have among the largest brain‐to‐body size ratios of any mammals (second only to humans).^108^ A second plausible contributor is our unusually long post‐reproductive lifespan, a feature shared almost exclusively by humans and toothed whales.^109^ Consistent with this, odontocetes are also among the few non‐human animals known to undergo menopause.^110^ This convergence raises the possibility that menopause‐related hormonal shifts may interact with neuromodulatory vulnerability, potentially helping to explain the higher incidence of AD in women^111^ and fitting established links between estrogen and cholinergic function.^112^

Together, these observations hint that AD may emerge where evolutionarily conserved neuromodulatory systems are pushed beyond originally selected limits to modulate much broader regions across significantly longer lifetimes. Comparative studies of neuromodulatory systems across humans, non‐human primates, and odontocetes could provide powerful opportunities to test the proposed framework.

## 9 CONCLUSIONS

We have proposed a neuromodulatory fragility hypothesis for AD pathogenesis. The proposed model offers a parsimonious account of how a single point of vulnerability (neuromodulatory projection neurons) can give rise to downstream hallmarks of AD, including the emergence of tau neurofibrillary tangles, Aβ plaques, neuroinflammation, cognitive–behavioral symptoms, and dementia onset. The key insight of this model is that neuromodulatory system dysfunction is inherently tied to, and sufficient to explain, both tau hyperphosphorylation and increases in neocortical Aβ, the two hallmarks of AD. Given the inherent fragility of these volume transmission projection neurons in humans, it is no surprise through the lens of this framework that AD has such high global prevalence.

In this framework, genetic and environmental risk factors modulate AD risk through their cumulative effects on neuromodulatory resilience. The model predicts that strengthening these systems therefore represents the most effective strategy for preventing AD. Conversely, treatments that target downstream pathology are expected to offer only modest benefit once neuromodulatory dysfunction is established.

Our ideas and conclusions complement and expand upon recently proposed priorities for AD research from the Neuromodulatory Subcortical Systems Professional Interest Area of the Alzheimer's Association International Society to Advance Alzheimer's Research and Treatment.^17^ By describing this hypothesis, we hope to further raise awareness of the importance of neuromodulatory subcortical systems in early AD, while providing a testable mechanistic explanation of pathogenesis that can help guide future research questions and the development of a new generation of effective disease‐modifying therapies.

## CONFLICT OF INTEREST STATEMENT

The authors declare no conflicts of interest. Author disclosures are available in the supporting information.

## Supporting information



Supporting Information
